# Evaluation of medical practices in oncology in the context of the COVID‐19 pandemic in France: Physicians’ point of view: the PRATICOVID study

**DOI:** 10.1002/cam4.3503

**Published:** 2020-10-06

**Authors:** Carole Helissey, Anatole Cessot, Laurys Boudin, Emile Romeo, Caroline Prieux, Djamel Ghebriou, Antoine Schernberg, Noemie Grellier, Charlotte Joly, Olivier Bauduceau, Constance Thibault, Elodie Mamou, Gauthier Raynal, Sophie Serey eiffel, Hervé Le Floch, Damien Ricard, Laurent Brureau

**Affiliations:** ^1^ Clinical Research unit Military Hospital Begin Saint‐Mandé France; ^2^ Department of Medical oncology Clinique HARTMANN Neuilly‐sur‐Seine France; ^3^ Department of Medical oncology Military Hospital Sainte‐Anne Toulon France; ^4^ Department of Gastroenterology Military Hospital Percy Clamart France; ^5^ Department of Oncology Tenon University Hospital Institut Universitaire de Cancérologie AP‐HP. Sorbonne Université Paris France; ^6^ Department of Radiation Oncology Hôpital Tenon Paris France; ^7^ Department of Radiation oncology Hôpital Henri Mondor Créteil France; ^8^ Department of Medical Oncology Hôpital Henri Mondor Créteil France; ^9^ Department of Radiation Oncology Clinique HARTMANN Neuilly‐sur‐Seine France; ^10^ Department of medical oncology HEGP APHP.Centre Paris France; ^11^ Department of Urology Clinque Métivet Saint‐Maur‐des Fossés France; ^12^ Department of Pulmonology Military Hospital Percy Clamart France; ^13^ Department of Neurology Military Hospital Percy Clamart France; ^14^ CHU de Pointe‐à‐Pitre Univ Antilles Univ Rennes Inserm EHESP Irset (Institut de Recherche en Santé, Environnement et Travail) – UMR‐S 1085 Pointe‐à‐Pitre France

**Keywords:** cancer patient care, Pandemic COVID‐19, Physicians

## Abstract

The cancer population seems to be more susceptible to COVID‐19 infection and have worse outcomes. We had to adapt our medical practice to protect our patients without compromising their cancer prognosis. The national PRATICOVID study aims to describe the adaptation of cancer patient care for this population. We analyzed data from nine different institutions. The primary endpoint was to assess the prevalence of adapted patient care during the pandemic. The secondary endpoints were to describe the point of view of clinicians and patients during and after the pandemic. We analyzed 435 medical procedures between 9^th^ of March and 30^th^ of April. Because of the COVID‐19 pandemic, 47.6% of the outpatients received modified patient care. Twenty‐four percent of scheduled surgeries were postponed, or were performed without perioperative chemotherapy, 18.4% followed a hypofractioned schedule, and 57% had an adaptive systemic protocol (stopped, oral protocol, and spacing between treatments). Seventy percent of physicians used telemedicine. During this period, 67% of the physicians did not feel distressed taking care of their patients. However, 70% of physicians are worried about the aftermath of the lockdown, as regards future patient care. The PRATICOVID study is the first to assess modification of patient care in cancer outpatients during an epidemic. With this unprecedented crisis, physicians were able to adapt their practice in order to protect their patients against the virus while ensuring continuity of patient care. But physicians are worried about the aftereffects of the lockdown specifically in regard to care pathway issues.

## Graphical abstract

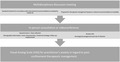

## 1 INTRODUCTION

In France, from the beginning of March 2020, 94,191 patients infected by severe acute respiratory syndrome coronavirus 2 (SARS cov2 or COVID‐19) were hospitalized and 25,561 patients died because of this new virus.^1^

Due to this public health emergency, our lives have changed and more demands have been put on our health‐care systems forcing them to reorganize.^2^

The elderly population and patients with comorbidities appear to develop severe forms of this disease. Furthermore, the cancer population seems to be more susceptible to infection and to have worse outcomes.^3^, ^4^, ^5^

Thus, clinicians have had to face two challenges in an unprecedented context: ensuring continuity of patient care for a disease that involves a life‐threatening prognosis while reducing patients’ vulnerability to this virus.

The aim of PRATICOVID, a French study, was to describe the adaptation of our medical and surgical management of cancer patients and clinicians’ point of view during this pandemic.

## 2 PATIENTS AND METHODS

PRATICOVID is a prospective multicenter observational study involving clinicians from nine sites in France. The study was declared to the National Institute for Health (*Institut National des Données de Santé*, *INDS*, *Data* MR3416230420) and was reported to the National Commission for Data Protection and Liberties (CNIL; reference number: 2217722v0). Data were analyzed and interpreted by the authors. All authors reviewed the manuscript.

### 2.1 Study design

Nine French hospitals took part in this study from March 9 to April 30. Three military hospitals, four university hospitals, and two private clinics. All of these are general hospitals, and have been actively involved in caring for patients infected with COVID‐19, with an increased number of beds in intensive care, infectious disease, internal medicine, and pulmonology.

These hospitals established dedicated care pathways for the management of patients infected with COVID‐19.

This prospective study was initiated 1 week before the health crisis and subsequent containment measures were declared at the national level.

Few recommendations for the management of patients were issued during this pandemic.

We evaluated the therapeutic management of cancer patients in these establishments.

**Figure 1:**
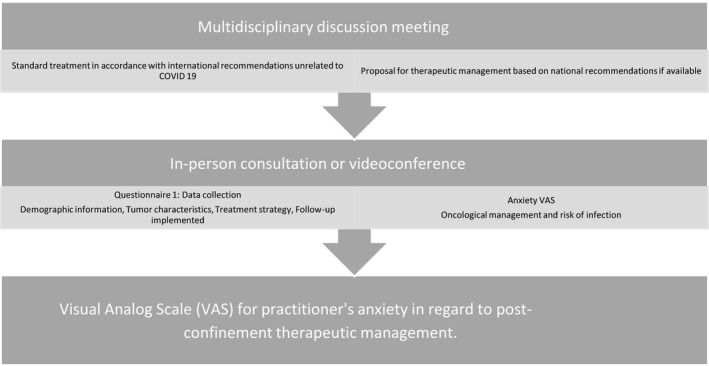
Study design

In the context of the pandemic, the therapeutic decision was debated in a multidisciplinary discussion meeting from which a therapeutic proposal was made. The proposal was then discussed with the patient and the management strategy was established (Figure 1).

**Figure 2:**
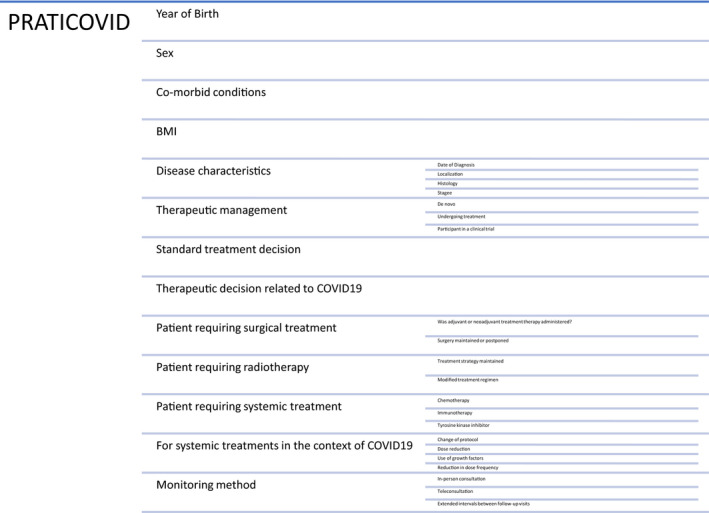
Questionnaire

Each investigator completed a questionnaire (Figure 2) for each patient seen in consultation or teleconsultation.

The first part was aimed at collecting demographic data (year of birth, sex, comorbidities, and body mass index), disease characteristics (primitive, histology, stage, and date of diagnosis), standard treatment, and treatment decision during the pandemic, type of usual follow‐up, type of follow‐up in the COVID‐19 context, type of treatment received, terms of treatment received, inclusion in a clinical trial or not.

The second part used an analog scale to assess practitioners’ and clinicians’ degree of anxiety with regard to therapeutic management in the context of the epidemic. Both the risk of reduced chances of successful cancer treatment and the risk of infection linked to COVID‐19 were taken into account.

The third part assessed practitioners’ degree of anxiety about post‐confinement oncological management and warning signs.

### 2.2 Study endpoints

The primary endpoint was to assess the prevalence of modified patient care during the pandemic. Modified patient care was defined as a postponed or canceled surgery, a postponed, canceled or modified irradiation protocol, a canceled or adapted systemic treatment or the use of telemedicine.

The secondary endpoints were to describe clinicians’ and patients’ points of view during and after the lockdown.

### 2.3 Statistical analysis

Body mass index was calculated as weight divided by height squared (kg/m^2^).

Differences between patient groups were assessed using the unpaired Student's t‐test and Mann‐Whitney U test for continuous covariates or the chi‐squared test for categorical covariates.

All statistical analyses were carried out with Statview software (SAS Institute, Cary, NC). All tests were two‐tailed, and *p* values <0.05 were considered significant.

## 3 RESULTS

### 3.1 Patients

From March 9 to April 30, 435 cancer patients were case‐managed at nine sites, by oncologists, surgeons, and radiation oncologists.

Among the main characteristics, the median age was 69 years (range, 24–99) and 53.6% were male (Table 1). In our cohort, 483 patients (65.1%) presented at least one comorbidity. Ninety‐seven percent of the patients had a solid tumor. There was a broad range of primary tumors including mainly breast (21.6%), prostate (20.7%), colorectal (14.7%), and lung (11.7%).

**Table 1:** Baseline characteristics

Variables	Without therapeutic changes	With therapeutic changes	All patients	*p* value
Number of patients (%)	228 (52.4)	207 (47.6)	435 (100.0)	**‐**
Gender N, (%)				
Female	100 (43.9)	102 (49.3)	202 (46.4)	.26
Male	128 (56.1)	105 (50.7)	233 (53.6)	
Age (years) median, (range)	69.0 (30.0–93.0)	71.0 (24.0–99.0)	69.0 (24.0–99.0)	.35
Age (years) N (%)				
≤65	95 (41.7)	75 (36.2)	170 (39.1)	.25
>65	133 (58.3)	132 (63.8)	265 (60.9)	
Age (years) N (%)				
≤50	25 (11.0)	26 (12.6)	51 (11.7)	
[50–60]	38 (16.7)	28 (13.5)	66 (15.2)	
[60–70]	66 (28.9)	47 (22.7)	113 (26.0)	.40
[70–80]	64 (28.1)	67 (32.4)	131 (30.1)	
> 80	35 (15.3)	39 (18.8)	74 (17.0)	
Body mass index (kg/m^2^) (*n*, %)				
<25	107 (57.0)	111 (61.3)	218 (59.0)	.56
25–30]	67 (35.6)	55 (30.4)	122 (33.1)	
>30	14 (7.4)	15 (8.3)	29 (7.9)	
New diagnosis				
No	143 (62.7)	125 (60.4)	268 (61.6)	.62
Yes	85 (37.3)	82 (39.6)	167 (38.4)	
Location of cancer				
Head and Neck	17 (7.5)	8 (3.9)	25 (5.7)	
Brain	4 (1.8)	2 (1.0)	6 (1.4)	
Lung	28 (12.3)	23 (11.1)	51 (11.7)	
Colorectal	26 (11.4)	38 (18.4)	64 (14.7)	
Prostate	56 (24.6)	34 (16.4)	90 (20.7)	
Breast	47 (20.6)	47 (22.7)	94 (21.6)	.21
Kidney	9 (3.9)	11 (5.3)	20 (4.6)	
Urothelial	13 (5.7)	16 (7.7)	29 (6.7)	
Gynecologic	13 (5.7)	12 (5.8)	25 (5.7)	
Hematology	6 (2.6)	3 (1.4)	9 (2.1)	
Others	9 (3.9)	13 (6.3)	22 (5.1)	
Comorbidities				
Cardiovascular	103 (53.1)	99 (55.9)	202 (54.4)	
Renal failure	10 (5.2)	8 (4.5)	18 (4.9)	.46
Chronic obstructive	11 (5.7)	8 (4.5)	19 (5.1)	
pulmonary disease	70 (36.1)	62 (35.1)	132 (35.6)	
Others				
Types of treatment				
Surgery	**19 (8.3** **)**	**6 (2.9)**	**25 (5.7)**	
Radiotherapy	**28 (12.3)**	**38 (18.4)**	**66 (15.2)**	**.003**
Systemic treatment	**150 (65.8)**	**118 (57.0)**	**268 (61.6)**	
Multimodal treatment	**31 (13.6)**	**45 (21.7)**	**76 (17.5)**	
Physicians’ feelings about COVID−19				
Not distressed	**63 (37.1)**	**71 (43.1)**	**134 (40.0)**	
Slightly distressed	**40 (23.5)**	**50 (30.3)**	**90 (26.9)**	**.04**
Somewhat distressed	**66 (38.8)**	**41 (24.8)**	**107 (31.9)**	
Very distressed	**1 (0.6)**	**3 (1.8)**	**4 (1.2)**	
Patients’ feelings about COVID−19				
Not distressed	**40 (25.2)**	**46 (27.7)**	**86 (26.5)**	
Slightly distressed	**40 (25.2)**	**59 (35.5)**	**99 (30.5)**	**.003**
Somewhat distressed	**78 (49.0)**	**53 (31.9)**	**131 (40.3)**	
Very distressed	**1 (0.6)**	**8 (4.9)**	**9 (2.7)**	
Centers				
Public practice	**43 (18.9)**	**48 (23.2)**	**91 (20.9)**	
Private practice	**56 (24.6)**	**74 (35.7)**	**130 (29.9)**	**.04**
Military hospital	**129 (56.5)**	**85 (41.1)**	**214 (49.2)**	

Bold values indicates Number of patient (%).

A total of 167 patients (38.4%) presented a new cancer diagnosis.

Sixty‐one percent of all patients were eligible for a systemic treatment, 15.2% for radiotherapy, 5.7% for surgery, and 17.5% for a multimodal treatment (chemotherapy with surgery and/or radiotherapy).

Two hundred and seven (47.6%) patients received modified patient care. In this group, the median age was 71 years (range, 24–99) and 50.7% were male. The main primary tumor site was breast cancer (22.7%) at a metastatic stage. Three percent of these patients were candidates for surgery, 18.4% for radiotherapy, 57% for a systemic treatment, and 21.7% dedicated to multimodal treatment. Seventy percent of patients were followed by telemedicine.

Four percent of all population were included in a clinical trial.

In the surgery cohort, 24% of the patients had a postponed surgery or did not receive perioperative chemotherapy.

In the radiotherapy group, 58% of the patients received a hypofractionated regimen.

In the systemic treatment cohort, 44% of the patients were given adapted patient care. Forty‐eight percent had an oral chemotherapy protocol, 46% had an interruption or cuts in immunotherapy.

Significant differences (*p* < 0.05) between groups with or without therapeutic adaptations were found with respect to treatment type (surgery, radiotherapy, systemic, and multimodal), institution type (public, private, and military), and both physicians’ and patients’ level of concern about COVID‐19.

### 3.2 Clinicians’ and patients’ views

**Figure 3:**
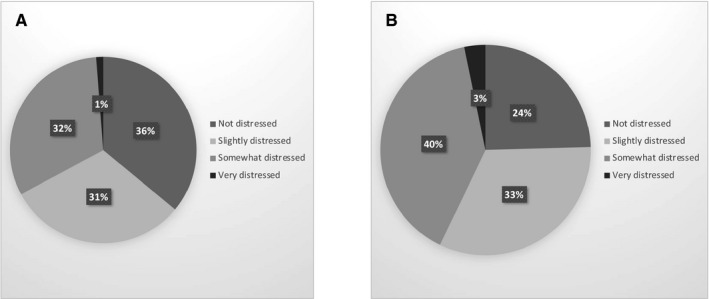
A. Physician’s view on patient care during the pandemic. B. Patient’s view on patient care during the pandemic

We obtained clinicians and patient's point of view on 411 patient treatments. Sixty‐seven percent of these procedures were judged hardly distressful during the peak of the pandemic (Figure 3).

**Figure 4:**
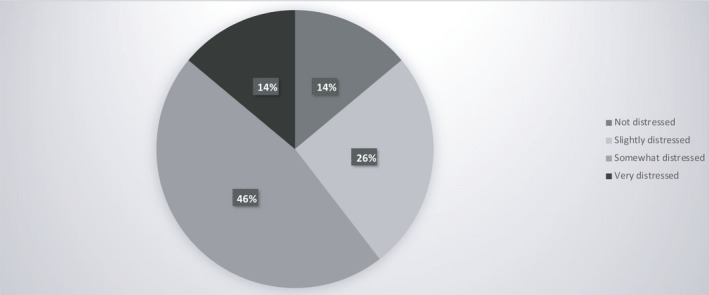
Physicians’ point of view on patient care after lockdown

**Figure 5:**
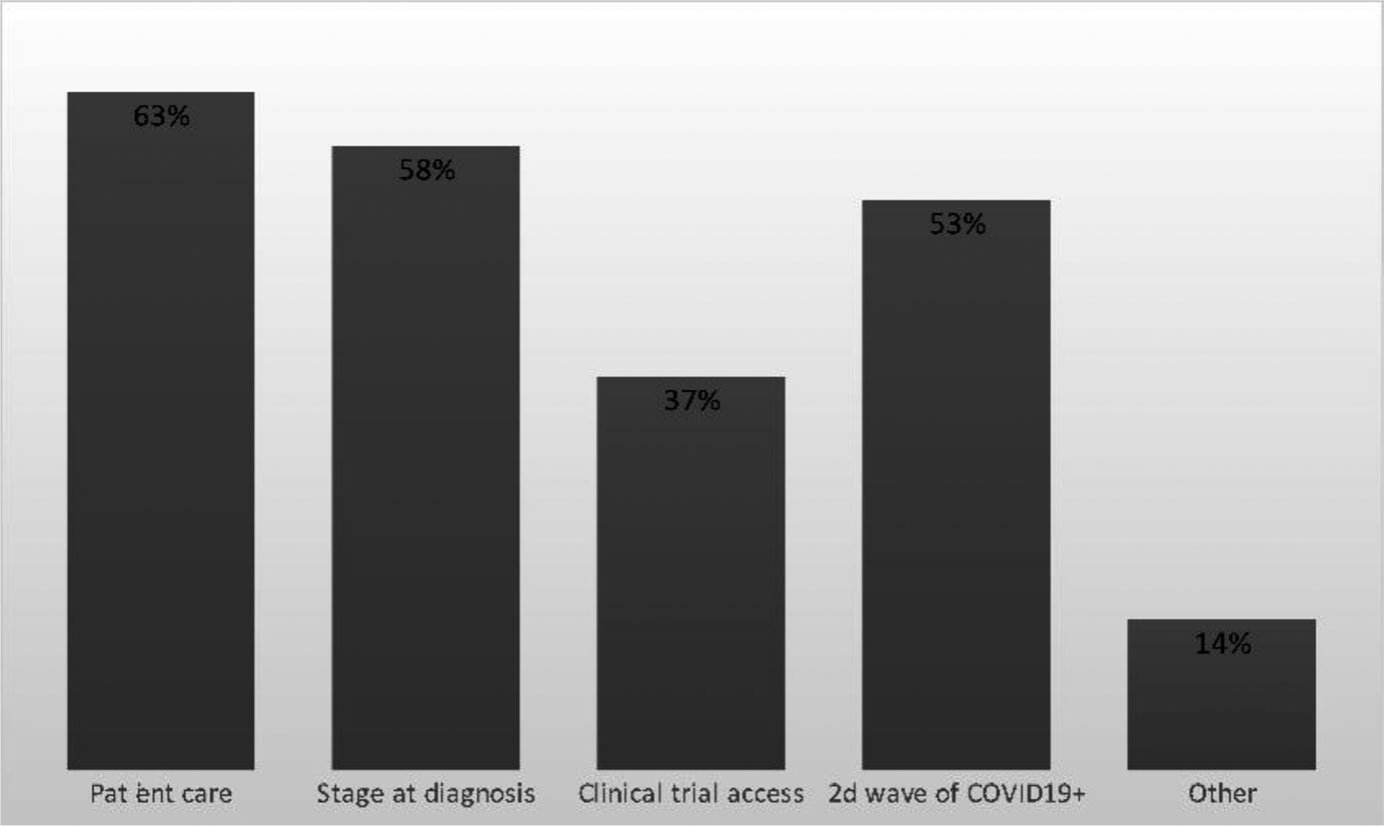
Physicians' point of view on alerted points

From a sample of 43 physicians (Table 2), we collected point of view data on patient care after lockdown. Sixty‐one percent are worried specifically about the organization of patient care (Figures 4 and Figure 5).

**Table 2:** Baseline characteristics of physicians

Main criteria	
*N*	43
Age (years), median (Range)	36 (range, 30–49)
Practice (*N*, %)	
Surgeon	13 (30%)
Oncologist	22 (51%)
Radiation Oncologist	8 (19%)
Year of practice (*N*, %)	
<5 years	12 (28%)
5–10 years	20 (47%)
>10 years	11 (26%)

## 4 DISCUSSION

Coronavirus disease 2019 (COVID‐19) has changed our lifestyles, and put a heavy strain on our health‐care systems.

At the peak of the pandemic, clinicians had to face the following dilemma: not expose their patients to the virus but maintain the best care of cancer patients.^6^, ^7^ COVID‐19 has turned conventional care upside down.

PRATICOVID is the first prospective study to analyze the impact of the pandemic on our medical care and our vision of future patient care. However, it raises very serious challenges for the future.

In France, from March to April, our joint effort was focused on clinical management of patients infected by COVID‐19. Physicians had to propose alternative forms of medical care including changes in protocol and the use of communication technologies to ensure follow‐up.

In our study, almost 48% of patients received modified care.

The main factors influencing the therapeutic modifications were treatment type, institution type, and both physicians’ and patients’ level of concern about COVID‐19.

In order to minimize immunosuppression and hospital admissions, 44% of patients underwent therapeutic de‐escalation in accordance with official recommendations. These included encouraging oral protocol, spacing out the treatment, and postponing the initiation of treatment in certain cases.^8^, ^9^ What impact these changes may have on the prognosis remains to be determined, however, and will surely affect decisions about maintaining or modifying protocols in the future. Given that patients have to go to their pharmacy to obtain their treatment, even more so with the amplification of oral protocols, reducing the risk of COVID‐19 infection is contingent on organizing patient care in pharmacies as well.

Once started, radiation therapy must not be stopped and patients have to come at the outpatient clinic 5 days a week. This could increase COVID‐19 exposure. Fifty‐eight percent of patients in our study received a hypofractionated schedule. This schedule has demonstrated its benefit in curative and palliative treatment,^10^, ^11^, ^12^, ^13^ and it is a good alternative to decrease the number of patient visits to the hospital.

Twenty‐four percent had postponed surgery and canceled perioperative chemotherapy. This decision was taken in order to minimize patients’ immunosuppression and according to the availability of places in intensive care. For selected patients, delaying surgery does not impact prognosis in cases such as stage I or II breast cancer or low intermediary risk prostate cancer.^8^, ^14^, ^15^ In other cases, however, this delay could impact the prognosis and the recurrence risk. Future studies are needed to address this question.

Telemedicine has demonstrated that it improves follow‐up and decreases health costs.^8^, ^16^ Our study highlights the extension of telemedicine. Indeed, 70% of the patients were followed by telemedicine. Telemedicine has clearly established itself in our care pathways and this is probably a long‐term development. However, there are issues which can limit its use in the future.

The major one is the limitation of physical exams.^6^ In France, telemedicine is reimbursed, but this is not the case in all health‐care systems or with all forms of health insurance. Furthermore, it requires the implementation of specific technological means to ensure both communication quality and confidentiality.

These strategies aim at minimizing nosocomial infections and reducing the spread of COVID‐19 infection in keeping with recommendations and previous reported experiences.^9^, ^17^, ^18^, ^19^

Human and material resources of each hospital are important factors that influenced the treatment strategy during this pandemic. In military hospitals, two independent patient care strategies were established, COVID+ and COVID‐ with two different teams of caregivers.

Our findings reveal that therapeutic adaptations are associated with lower levels of concern about COVID‐19 among both physicians and patients. New approaches to management must reduce the risk of exposing patients to COVID‐19. The potentially increased risk of COVID‐19 infection appears to be a significant source of concern in the group of physicians and the patients without therapeutic changes (49%) and may be an inhibiting factor.

However, our study raises some concerns about the patients’ prognosis.

In our overall population, only 38.4% of patients were newly diagnosed. Similarly, Dinmohamed et al. reported a notable decrease in cancer diagnoses, as much as 27%.^20^ In the Netherlands, during this period, patients preferred not to consult their general practitioners if they did not present symptoms of COVID‐19. Furthermore, screening programs have been suspended. Presumably, therefore, many new cancer cases have gone undiagnosed during the COVID‐19 pandemic, due in part to patients’ reluctance to consult their physician and preoccupation with COVID‐19. This creates a further source of apprehension for practitioners about the stage of disease when the eventual diagnosis is made, the impact on survival,^21^ and whether our hospitals will be able to accommodate a surge in the number of new cancer cases after the pandemic. We must encourage patients to consult their doctor and screening programs must be resumed immediately.

Moreover, only 4% of the patients were included in a clinical trial. Waterhouse et al. reported the early impact of COVID‐19 on the conduct of oncology clinical trials. Sixty percent of clinicians stopped screening, enrollment, and research‐only visits except those providing cancer treatment. The majority reported ceasing research‐only blood and/or tissue collections. They reported different issues like decrease in patient ability or willingness to come to their site and limited availability of ancillary services (e.g., radiology, surgery, cardiology, etc.).^21^

Modified medical care for all patients could worsen the prognosis. In our study, 52.4% of patient treatments did not change. Physicians judged that cancer prognosis was more important than a potential risk of COVID‐19 infection.

Despite the unexpectedness of the situation, the clinicians were not distressed taking care of their patients (67%). Many patients were in doubt as to how COVID‐19 might affect their care^6^ (57%). Cancer prognosis could be perceived more negatively than COVID‐19 infection.^22^ This highlights the importance of discussion between oncologists and patients.

Physicians are concerned about the future and clear guidelines must be established. Decisions must be made as to whether all patients should be screened for COVID‐19, whether treatment should be suspended for asymptomatic COVID‐19 patients and for how long, how patients should be recruited for clinical trials, and what sort of follow‐up is most appropriate. The mental and physical well‐being of caregivers are factors that must also be taken into consideration, as well as their capacity to cope with a probable surge in new patients especially in the event of a second wave.

Faced with this unprecedented crisis, physicians were able to adapt their practice with the first goal of protecting their patients against the virus while ensuring the course of patient care. Telemedicine in particular seems destined to play an important role. But now is the time to take further steps. A new challenge arises which entails facing not only a possible second wave of patients infected with COVID‐19 but also a wave of cancer patients who have not been diagnosed or received care during the pandemic confinement.

Will this be an opportunity to rethink the patient care process and the conduct of clinical trials? However it may be, we face this new challenge with confidence and high hopes.

## CONFLICT OF INTEREST

The authors declare that they have no conflict of interest.

## Data Availability

The data that support the findings of this study are available on request from the corresponding author. The data are not publicly available due to privacy or ethical restrictions.
